# Profiling of blood miRNAomes revealed the potential regulatory role of miRNAs in various lameness phenotypes in feedlot cattle

**DOI:** 10.1186/s12864-024-10807-z

**Published:** 2024-12-18

**Authors:** Zhe Pan, Wentao Li, Sonja Bialobzyski, Yanhong Chen, Eoin O’Hara, Hui-zeng Sun, Karen Schwartzkopf-Genswein, Le Luo Guan

**Affiliations:** 1 Department of Agricultural, Food and Nutritional Science, University of Alberta, Edmonton, AB T6G2P5 Canada; 2 Faculty of Land and Food Systems, The University of British Columbia, Vancouver, BC V6T 1Z4 Canada; 3 Agriculture and Agri-Food Canada, Lethbridge, AB T1J 4B1 Canada; 4 Institute of Dairy Science, College of Animal Sciences, Zhejiang University, Hangzhou, 310058 China

**Keywords:** Lameness, Foot rot, Digital dermatitis, Toe tip necrosis, Blood miRNAs, MiRNAomes, Diagnostic markers

## Abstract

**Background:**

Lameness is a collective term for multiple foot diseases in cattle including, but not limited to, foot rot (FR), digital dermatitis (DD), and toe tip necrosis (TTN), which is a critical welfare concern. The diagnosis of specific phenotypes of lameness in feedlot cattle is challenging and primarily relies on visual assessments. However, different lameness phenotypes share similar clinical symptoms and there is a limited understanding of potential biomarkers relating to such disease for further molecular diagnosis. This study aimed to identify blood miRNA profiles of feedlot cattle with various lameness phenotypes and whether they could be potential diagnostic markers to differentiate lameness phenotypes and predictive lameness recovery.

**Results:**

MicroRNAome profiles were generated for the whole blood samples collected from feedlot cattle at Week 0 (W0) before treatment (*n* = 106) and longitudinal miRNA expression profiles relating to lameness recovery from W0 to W2 (*n* = 140) using RNA-seq. Ten miRNAs were selected to verify miRNA sequencing accuracy using stem-loop RT-qPCR. A total of 321 miRNAs were identified to be expressed in bovine blood samples with three (all downregulated, average log_2_fold change = -1.32), seven (two downregulated with average log_2_fold change = -1.15, five upregulated with average log_2_fold change = 1.68), six (three downregulated with average log_2_fold change = -1.23, three upregulated with average log_2_fold change = 3.31), and fourteen (eight downregulated with average log_2_fold change = -1.24, six upregulated with average log_2_fold change = 1.26) miRNAs differentially expressed (DE) miRNAs in DD, FR, TTN, and FR combined with DD (FRDD) compared to healthy control at W0 (defined as pre-treatment DE miRNAs), respectively. The predicted functions of identified DE miRNAs among different lameness phenotypes were mainly related to Zinc-finger, muscle cell development, and host inflammatory responses. Furthermore, the longitudinal miRNA expression profiles revealed that a total of eight miRNA changed patterns from W0 to W2, with the BTB/POZ-like domain being the most enriched function by longitudinal miRNA expression profiles in both unrecovered and recovered cattle. A total of nine miRNAs (five downregulated with average log_2_fold change = -2.4, four upregulated with average log_2_fold change = 3.7) from W0 to W2 were differentially expressed in unrecovered cattle compared to the recovered cattle, with functions associated with transcription regulation and Zinc-finger. Moreover, the area under the receiver operating characteristics (ROC) curve (AUC) revealed that pre-treatment DE miRNAs could serve as good diagnostic markers to differentiate any two of four phenotypes of lameness, with bta-miR-339b being able to differentiate all lameness phenotypes. Moreover, pre-treatment DE miRNAs could also predict the recovery of three lameness phenotypes (DD, FRDD, TTN) with good to excellent predictiveness.

**Conclusion:**

Our results comprehensively assessed the blood miRNAomes in response to various lameness phenotypes, promoting the understanding of miRNA-regulated mechanisms of lameness in feedlot cattle. The diagnostic miRNA markers were also identified to differentiate within lameness phenotypes and predictive lameness recovery, shedding light on accurate on-farm lameness detection.

**Supplementary Information:**

The online version contains supplementary material available at 10.1186/s12864-024-10807-z.

## Background

Lameness, a collective term for multiple foot diseases, is a significant economic and welfare concern for cattle because it can lead to reduced water and food intake, pain, and decreased production performance [1–4]. The prevalence of lameness is high in feedlot cattle, which has been reported to be up to 32% in Canadian farms based on a ten-year study conducted across 28 feedlots [3, 5]. Lameness can have different clinical phenotypes including foot rot (FR), digital dermatitis (DD), and toe tip necrosis (TTN), each with distinct diagnostic criteria and specific treatments [6–8]. The current lameness detection approach primarily relies on visual assessments, which evaluate clinical signs such as arched backs, impaired movements, abnormal gaits, and foot lesions [3, 9–11]. However, this observational diagnosis is subjective, requires the professional training of staff, and requires evident locomotion abnormalities in feedlot cattle [12]. Recently, automated lameness detection systems (ALDS), such as automatic deep image analysis and accelerometer systems, have been proposed for the detection of lameness [9, 12]. Nonetheless, these techniques require frequent, uninterrupted inspections (at least twice a week) and are designed primarily for dairy cows [9, 12] and thus are not practical for feedlot cattle. Additionally, clinical signs of different lameness types during the early stage are similar, and therefore, proper treatments are unlikely to be used for the corresponding lameness phenotype, which can lead to increased recovery time [12, 13]. The previous lameness studies mainly focused on dairy cattle with digital dermatitis and reported altered expressions of genes involved in inflammation such as *A2ML1*, *PI3*, *CCL11* and the most downregulated genes with functions of keratins (such as *SCGB1D*) and anti-inflammatory molecules that activate pro-inflammatory IL-17 signaling pathway (such as *MGC151921*) [1]. Consequently, a precise and convenient diagnostic approach is needed to facilitate effective on-farm detections of lameness phenotypes for feedlot beef cattle.

Recent studies have revealed that certain biological molecules, such as microRNAs (miRNAs), can be utilized as diagnostic markers to identify diseases and health conditions of interest [14, 15]. These miRNAs are effective diagnostic markers for both human and animal diseases [16]. The miRNAs are approximately 21 ~ 25 nucleotide non-coding RNAs expressed in various cells, tissues, and body fluids with functions of down-regulating host gene expressions through the degradation of target messenger RNA (mRNA) and translation inhibition [16]. Notably, the expression levels of circulating miRNAs (miRNAs that circulate in body fluids) are responsive to a range of host physiological conditions. For instance, bta-miR-1976, bta-miR-873-3p, and bta-miR-520f-3p were differentially expressed in the serum of cattle with Johne’s disease compared to healthy cattle, serving as potential markers for diagnosis [17]. Likewise, differentially expressed miRNAs in the blood were reported in heat-stressed dairy cows (*i.e.* bta-miR-19a and bta-miR-2284a), suggesting that they play a critical role in regulating the heat stress response, and may also act as potential diagnostic markers for detecting heat stress incidents [18]. Furthermore, previous studies reported that expressions of genes (*i.e. Interleukin-10*, *matrix metalloproteinase-13*) in blood cells (*i.e*. leukocytes, mononuclear cells) of lame dairy cattle differed from those in healthy controls [19, 20], thus providing a molecular basis for identifying the role of miRNAs in lameness.

In this study, we ​hypothesized that there are distinct differences in blood miRNA profiles between healthy cattle and those displaying various lameness phenotypes, and these differences are associated with the development of lameness. We also speculated that the patterns and functions of blood miRNA profiles in cattle that had recovered or not recovered from the aforementioned lameness phenotypes are different and phenotype-specific, and differentially expressed miRNAs identified from lame cattle pre-treatment could be effective predictive biomarkers for recovery. Our objective was to identify differentially expressed and phenotype-specific miRNAs in the blood of lame cattle and to uncover the dynamic changes in miRNA patterns over time in response to lameness recovery or non-recovery. The findings from this study will contribute to an improved understanding of the potential of miRNAs as diagnostic markers for lameness and the mechanisms of miRNAs participating in lameness pathogenesis.

## Materials and methods

### Whole blood sample collection and lameness phenotype identification

Cattle used in this study were from a commercial feedlot in Southern Alberta. The classification of different lameness phenotypes was determined based on visual assessments of lesions, together with M-stage scoring and gait scoring, conducted by trained technical staff at the Lethbridge Research and Development Centre (Lethbridge, AB). Specifically, the gait score of each lame animal was categorized according to a 4-point integral scale (from 0 to 3) previously described by Webster et al. (2008), with 0 defined as no lameness, 1 (imperfect mobility with shortened strides and uneven steps), 2 (impaired mobility with immediately identifiable uneven weight-bearing on a limb and/or obviously shortened strides) and 3 (severely impaired mobility with symptoms such as arched back) defined as the most severe lameness. Causes of lameness were assigned to different diagnoses, such as digital dermatitis (DD), toe tip necrosis (TTN), foot rot (FR), and digital dermatitis combined with foot rot (FRDD). According to the diagnosis, animals were subjected to phenotype-specific treatments using various treatments based on individual feedlot protocols.

**Fig. 1:**
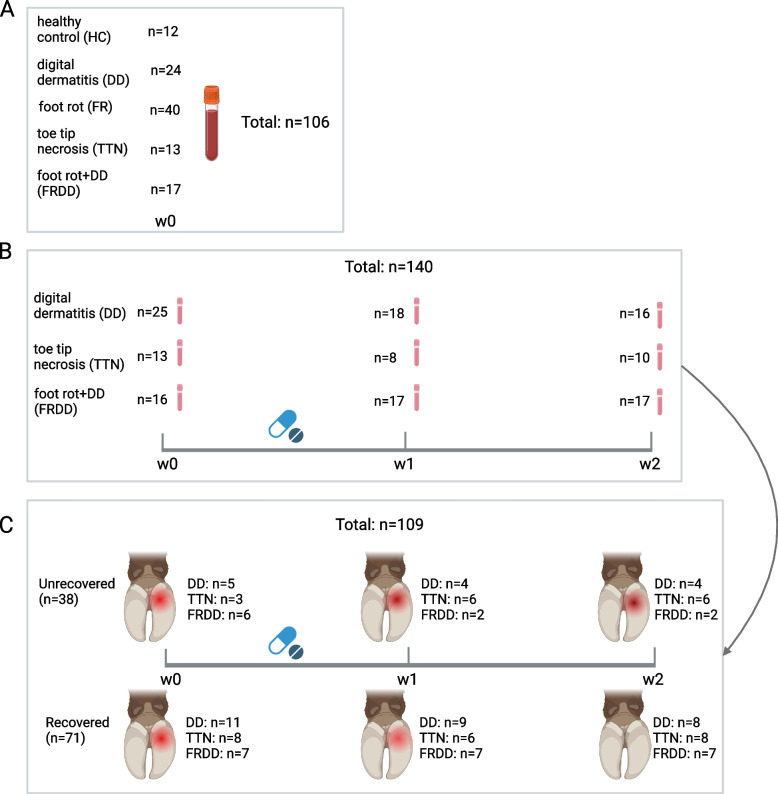
The schematic diagram of sample collection from feedlot cattle. **A **The whole blood sample collection in feedlot cattle with DD, FR, TTN, and FRDD and healthy control pre-treatment. **B **The whole blood sample collection in feedlot cattle with DD, TTN, and FRDD from W0 to W2. **C** The whole blood sample collection for recovered and unrecovered feedlot cattle with DD, TTN, and FRDD from W0 to W2

Whole blood samples were collected using venipuncture and Tempus™ Blood RNA Tubes (Thermo Fisher Scientific, CA) from 106 cattle without treatment (Week 0, W0), including DD (*n* = 24), FR (*n* = 40), TTN (*n* = 13), FRDD (*n* = 17), as well as healthy controls (HC, *n* = 12, Fig. 1A). In addition, 140 whole blood samples were collected throughout the experiment, using the same approach, from cattle diagnosed with three lameness phenotypes including DD (*n* = 59), TTN (*n* = 31), and FRDD (*n* = 50). These samples were collected at three time points: initial diagnosis and before treatment (Week 0, W0), one week after diagnosis and treatment (Week 1, W1), and two weeks after diagnosis and treatment (Week 2, W2, Fig. 1B).

### Total RNA extraction and quality assessment

Total RNA was extracted from whole blood samples using Preserved Blood RNA Purification Kit I (Norgen, Thorold, ON, CA) following the manufacturer's protocol. The Tempus™ Blood RNA Tubes were thawed at room temperature and the entire contents were poured into a new 50 mL conical tube. Samples were then mixed with 3 mL of Tempus™ Blood RNA Tube Diluent and centrifuged at 4 °C at 5000 × g for 30 min. Then, the separated RNA pellet was lysed using the provided Lysis Solution. Three hundred (300) μL of ethanol was added to allow all contents to be transferred to the Mini Spin column, which was followed by two washes using the provided Washing Solution. The RNA pellet was then dissolved in 50 μL Elution Solution.

The concentration and quality of total RNA was determined using UV spectrum and absorbance on a NanoDrop ND-2000 spectrophotometer (Thermo Fisher Scientific, Wilmington, DE) by the ratio of A260/A230 and A260/A280. RNA Integrity Number (RIN) of the total RNA was further assessed to determine the RNA quality and integrity using a 2200 TapeStation Instrument with RNA ScreenTapes (Agilent Technologies, Santa Clara, CA), and only RNA that passed the quality control with RIN > 7 were subjected to the miRNA library construction.

### MiRNA RNA-seq library construction and miRNA sequencing

The total RNA (1.0 μg) extracted from the whole blood samples was used for miRNA sequencing library construction using QIAseq miRNA Library Kit (96) (Qiagen, CA) following the manufacturer’s instructions. Briefly, the total RNA was first subjected to the 3’ and 5’ adapter ligation to increase the nucleic acid fragment length (3’ adapter: AACTGTAGGCACCATCAAT; 5’ adapter: GTTCAGAGTTCTACAGTCCGACGATC). Then, the reverse transcription of the ligated small RNA sequence was carried out, using the provided reagents, on a GeneAmp PCR System 9700 (Invitrogen, Carlsbad, CA, USA) with the following steps: 50 ºC incubation for one hour, 15 min at 70 ºC, and then held at 4 ºC until further PCR amplification. PCR enrichment was then performed using the following steps: Held at 95 ºC for 15 min, followed by 11 cycles of 95 ºC for 15 s, annealing at 60 ºC for 30 s. Extension at 72 ºC for 15 s, then held at 72 ºC for 2 min. Enriched cDNA libraries were then cleaned up using QIAseq miRNA Next Generation Sequencing (QMN) Beads (Qiagen, CA) on a magnetic stand and quantified on a Qubit 2.0 Fluorometer (Invitrogen, Carlsbad, CA). The cDNA library was then sequenced at Génome Québec (Montréal, Canada) using an Illumina HiSeq 2000 system (Illumina) with 50 base pair single reads.

### miRNA sequence data analysis

The miRNA sequencing data were subjected to a web-based tool, sRNAtoolbox (https://arn.ugr.es/srnatoolbox/), for quality control, adapter trimming, and read length distribution analysis. Reads that passed the quality control with lengths larger than 15 nt were aligned to the bovine miRNA database (miRBase v22.2, http://www.mirbase.org/) to identify known bovine miRNAs using the default parameter (alignment type: bowtie seed alignment; seed length for alignment: 20; minimum read count: 2; minimum read length: 15; allowed number of mismatches: 2; the maximum number of multiple mappings: 10). The mapped counts were then normalized to RPM (Reads per million mapped reads), with RPM > 1 considered as expressed.

### Identification of differentially expressed and phenotype-specific miRNAs

The expression level of miRNAs was compared between healthy cattle and three lameness groups at W0, and among various lameness phenotypes at W0 to identify differentially expressed miRNAs (DE miRNAs) before treatment using the DESeq2 package in R [21]. Additionally, DE miRNAs that were associated with lameness recovery patterns were also identified for each lameness phenotype from W0 to W2 using the same R package. The DE miRNAs were considered as expressed miRNAs with absolute log_2_ fold change > 1 and false discovery rate (FDR) < 0.05. The miRNAs expressed in at least 60% of samples within one lameness phenotype but not in other groups were considered phenotype-specific expressed miRNAs. The expressed miRNAs that were only identified at one time-point in each lameness phenotype were considered time-point-specific miRNAs.

### Assessment of dynamic expression patterns of miRNA profiles in response to lameness recovery

Cattle initially diagnosed with lameness at W0 and with at least one gait score (≥ 1) recorded at W1 or W2 indicating a trend of recovery, or not recovered, were further selected to investigate the role of miRNAs for lameness recovery (*n* = 109, Fig. 1C). The Mfuzz package in R was adopted to identify the dynamic changing patterns of miRNA expressions between recovered and unrecovered cattle for three lameness types during the three-week treatment period. The Mfuzz is a fuzz C-Means clustering (FCM) method [22] and is capable of clustering expressed miRNAs based on the similarities of longitudinal miRNA expressions. The miRNA expression clusters were identified based on the average RPM of each miRNA. For each identified cluster, a miRNA with a membership score > 0.7 was considered a signature miRNA.

### Assessment of predictiveness of selected miRNAs for lameness phenotype differentiation and lameness recovery

The identified DE miRNAs among various lameness phenotypes pre-treatment were used to assess their diagnostic power to differentiate lameness phenotypes. Additionally, the phenotype-specific and differentially expressed miRNAs pre-treatments were selected to evaluate their predictiveness of lameness recovery from W0 to W2. The area under the ROC curve (AUC) was calculated using the pROC package in R to assess the power for lameness phenotype differentiation and the predictiveness of each miRNA for lameness recovery among three time points. The criteria for AUC were excellent (0.9–1), good (0.8–0.9), fair (0.7–0.8), weak (0.6–0.7), or fail (0.5–0.6) [23, 24].

### Prediction of targeted genes and functions of miRNAs

The target genes prediction for DE miRNAs, phenotype-specific miRNAs, and signature miRNAs from time-series changing patterns was performed using TargetScan (Release 7.2). The potential functions of selected miRNAs were predicted using Database for Annotation, Visualization and Integrated Discovery (DAVID) [25]. Predicted functions were clustered and sorted based on their correlation level (Enrichment score) and filtered by a threshold of FDR < 0.05.

### Validation of differentially expressed miRNAs expression using RT-qPCR

Several miRNAs were selected for the verification of miRNA sequencing accuracy using stem-loop RT-qPCR with Taqman miRNA assays (Applied Biosystems, Carlsbad, CA). Two internal standards, bta-miR-93 and bta-miR-16b, were selected based on previous bovine blood miRNA research results published by our group and were the most universally expressed miRNAs from the sequencing results [26, 27]. The relative expression level of each target miRNA was calculated by the ΔΔCt method. One-way ANOVA was used to compare the miRNA expression differences with *p* < 0.05 being considered as the significant difference.

## Results

### Blood miRNA profiles in lame and healthy beef cattle

**Fig. 2:**
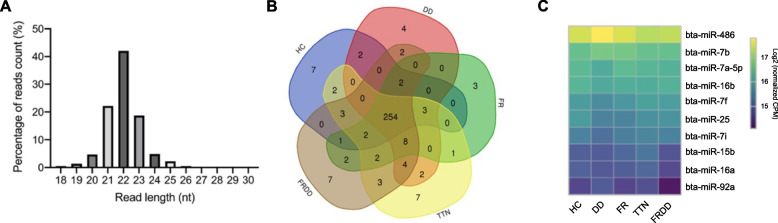
Overview of miRNAs expressed in blood samples collected from feedlot cattle using miRNA sequencing. **A **Length distribution of identified small RNA reads from blood samples collected from beef steers generated from RNA-seq. **B **Comparisons of the number of expressed miRNAs detected in HC, DD, TTN, FR, and FRDD. Colors represent different groups. **C **The expression of the top 10 highly expressed miRNAs in HC, DD, TTN, FR, and FRDD. Colors represent RPM as indicated by the color bar

A total of 709 million reads were obtained for the RNA-seq of whole blood samples collected from healthy and lame cattle at W0 with a distribution of reads length mostly ranging between 21–23 nt (Fig. 2A). The top 10 expressed miRNAs accounted for more than 80% of all reads. Among them, bta-miR-486 was the most expressed miRNA followed by the bta-let-7 miRNA family (Fig. 2C). A total of 321 known miRNAs were identified as expressed miRNAs from all blood samples at W0, with 273 in HC, 280 in DD, 289 in TTN, 280 in FR, and 286 in FRDD. For the expressed miRNAs, 254 out of 321 miRNAs were shared among all samples. Specific miRNAs were designated as DD- (4), FR- (3), TTN- (7), FRDD- (7), and healthy- (7) (Fig. 2B) with the expression level ranging between 1.16 ± 0.38 and 7.46 ± 6.20 RPM (Table 1). In addition, a total of 1,212 million reads were obtained for miRNAomes from 140 whole blood samples with size distribution mainly ranging from 21–23 nt between W0 and W2. Among identified miRNAs from W0 to W2, 307, 318, and 204 miRNAs were expressed in DD, TTN, and FRDD, respectively (Fig S1). Expression of 267, 277, and 273 miRNAs was maintained throughout the experiment (W0 to W2) in DD, TTN, and FRDD, respectively (Fig S1).

**Table 1:** The expression levels of phenotype-specific miRNAs

	Phenotype specific miRNA	Average expression level (RPM)
HC	bta-miR-10b	1.28 ± 0.47
	bta-miR-2903	1.78 ± 0.76
	bta-miR-2285aj-5p	1.48 ± 0.64
	bta-miR-10164-3p	1.70 ± 0.88
DD	bta-miR-2887	3.04 ± 3.49
	bta-miR-2320-3p	1.28 ± 0.60
	bta-miR-2904	7.46 ± 6.20
TTN	bta-miR-7861	1.16 ± 0.38
	bta-miR-11998	1.60 ± 0.53
	bta-miR-483	2.91 ± 2.30
	bta-miR-2285dj	1.26 ± 0.69
FR	bta-miR-2382-5p	1.56 ± 0.80
	bta-miR-200a	3.60 ± 2.82
FRDD	bta-miR-1388-5p	1.28 ± 0.66
	bta-miR-494	1.69 ± 1.01
	bta-miR-2483-5p	1.34 ± 0.72

### Identification and functional enrichments of differentially expressed miRNAs in cattle with diverse lameness phenotypes before treatment

In total, three, seven, six, and fourteen miRNAs were identified as differentially expressed (DE) miRNAs in DD, FR, TTN, and FRDD compared to HC, respectively (Table 2). Among these DE miRNAs, bta-miR-6119-3p was the only DE miRNA down-regulated in all lameness phenotypes compared to HC (Table 2). Additionally, the expression of both bta-miR-133a and bta-miR-206 were upregulated in TTN and FR compared to HC (Table 2). However, the expression of bta-miR-340 and bta-miR-2408 were downregulated in TTN and DD compared to HC (Table 2). Similarly, the expression of bta-miR-125a was downregulated in FR and FRDD compared to HC (Table 2). A total of 5, 20, 6, 12, 7, and 15 DE miRNAs were identified between DD vs FR, DD vs FRDD, DD vs TTN, FR vs TTN, and FRDD vs TTN, respectively (Table S1).

**Table 2:** DE miRNAs and enriched functions between health control and cattle with different lameness phenotypes at W0

Group	DE miRNAs	log2Fold Change	P-adj	Functional Annotation Clustering	Functions
**DD vs HC**	bta-miR-6119-3p	-1.5	2.00E-04	Zinc-finger; Zinc ion binding; Zinc; Metal-binding	Formation of seborrhea-like dermatitis; Formation of psoriatic epidermis
	bta-miR-2408	-1.46	5.00E-04	Src homology-3 domain; CGMP-PKG signaling pathway; Zinc-finger; Zinc ion binding	Development of inflammatory disease
	bta-miR-340	-1.01	7.00E-03	Zinc-finger; Zinc ion binding; Zinc; Metal-binding	Inhibition of cancer cells growth
**FR vs HC**	bta-miR-6119-3p	-1.29	2.00E-04	Zinc-finger; Zinc ion binding; Zinc; Metal-binding	Formation of seborrhea-like dermatitis; Formation of psoriatic epidermis
	bta-miR-125a	-1.01	2.00E-04	Voltage-gated channel; Ion transport; Zinc finger, C2H2-like; Metal ion binding	Inhibition of cell cycle progression
	bta-miR-200c	1.14	1.00E-04	Serine/threonine-protein kinase, active site	Suppress tumor growth, cell migration and invasion
	bta-miR-30a-5p	1.17	6.00E-04	zinc ion binding; K Homology domain	Suppress tumor growth, cell migration and invasion
	bta-miR-10225a	1.31	4.00E-03	N/A	N/A
	bta-miR-206	1.81	4.00E-03	N/A	N/A
	bta-miR-133a	2.45	8.00E-04	N/A	N/A
**FRDD vs HC**	bta-miR-6119-3p	-1.75	3.00E-04	Zinc-finger; Zinc ion binding; Zinc; Metal-binding	Formation of seborrhea-like dermatitis; Formation of psoriatic epidermis
	bta-miR-1434-3p	-1.58	7.00E-03	N/A	N/A
	bta-miR-211	-1.23	2.00E-02	Cadherin-like; Cadherin conserved site; Sterile alpha motif/pointed domain	Host immune regulation
	bta-miR-1246	-1.09	3.00E-02	N/A	N/A
	bta-miR-125a	-1.09	3.00E-04	Voltage-gated channel; Ion transport; Zinc finger, C2H2-like; Metal ion binding	Inhibition of cell cycle progression
	bta-miR-484	-1.08	7.00E-04	N/A	N/A
	bta-miR-92b	-1.07	6.00E-05	MAD homology 1, Dwarfin-type; CTF transcription factor/nuclear factor 1	*E. coli *lipopolysaccharide-mediated inflammatory injury
	bta-miR-1306	-1.03	8.00E-03	N/A	N/A
	bta-miR-2378	1.03	4.00E-02	N/A	N/A
	bta-miR-338	1.04	9.00E-03	N/A	N/A
	bta-miR-497	1.12	3.00E-04	Protein kinase, catalytic domain; Serine/threonine-protein kinase, active site	Muscle traumatic stress
	bta-miR-210	1.17	1.00E-05	FoxO signaling pathway	Bovine early intramuscular adipogenesis
	bta-miR-345-5p	1.47	1.00E-09	N/A	N/A
	bta-miR-21-3p	1.71	2.00E-06	N/A	N/A
**TTN vs HC**	bta-miR-6119-3p	-1.36	7.00E-03	Zinc-finger; Zinc ion binding; Zinc; Metal-binding	Formation of seborrhea-like dermatitis; Formation of psoriatic epidermis
	bta-miR-2408	-1.22	2.00E-02	Src homology-3 domain; CGMP-PKG signaling pathway; Zinc-finger; Zinc ion binding	Development of inflammatory disease
	bta-miR-340	-1.12	1.00E-02	Zinc-finger; Zinc ion binding; Zinc; Metal-binding	Inhibition of cancer cells growth
	bta-miR-1	2.67	8.00E-03	DNA-binding	Regulate skeletal muscle development
	bta-miR-206	2.94	3.00E-05	SH3 domain; BTB/POZ-like	Regulate muscle cell differentiation
	bta-miR-133a	4.32	1.00E-07	Pleckstrin homology-like domain	Suppress tumor growth, cell migration and invasion

A total of 16 phenotype-specific and 22 DE miRNAs were subject to functional predictions. From the 22 DE miRNAs, only 13 exhibited significantly enriched functions (seven upregulated and six downregulated, Table 2). Four enriched functions using upregulated miRNAs (bta-miR-1 and bta-miR-206 in TTN, bta-miR-497 and bta-miR-210 in FRDD) were related to muscular functions including skeletal muscle development, muscle cell differentiation, muscle traumatic stress, and bovine early intramuscular adipogenesis, respectively (Table 2). Only one upregulated miRNA in FR, bta-miR-30a, was predicted to play essential roles in the Zinc-finger protein and zinc-iron binding functions (Table 2). Moreover, functions enriched using four downregulated DE miRNAs (bta-miR-340 and bta-miR-2408 in DD and TTN, bta-miR-125a in FR and FRDD, and bta-miR-6119-3p in all phenotypes) were related to Zinc-finger, while enriched functions of the other two downregulated DE miRNAs (bta-miR-211 and bta-miR-92b in FRDD) were associated with inflammatory and host immune responses (Table 2).

### Identification of differentially expressed miRNAs and enriched functions relating to lameness recovery

**Fig. 3:**
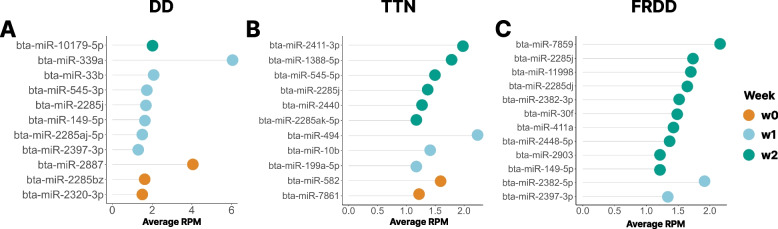
The lollipop plot showing time point-specific miRNAs in cattle with DD (**A**), TTN (**B**), and FRDD (**C**) from W0 to W2

A total of 30 miRNAs were identified as time-point specific miRNAs among each lameness phenotype (Fig. 3). Specifically, 11, 10, and 9 miRNAs were time point-specific miRNAs in DD, TTN, and FRDD, respectively (Fig. 3). Among these, bta-miR-2285j was identified as time point-specific miRNA in DD at W1 (Average RPM = 1.60 ± 0.93), TTN in W2 (Average RPM = 1.33 ± 0.68), and FRDD in W1 (Average RPM = 1.72 ± 1.45, Fig. 3). Furthermore, bta-miR-2397-3p was identified as time point-specific in both DD and FRDD at W1 (Average RPM_bta-miR-2397-3p_ = 1.21 ± 0.57 for DD at W1, 1.34 ± 0.45 for FRDD at W1, Fig. 3).

A total of 9 DE miRNAs were identified in unrecovered lame cattle compared to those that had recovered by W2 in all phenotypes (Table 3). Among these, bta-miR-1 was the only miRNA with the upregulated expression in unrecovered DD cattle at W0 (Table 3). Three miRNAs (two downregulated: bta-miR-2903 and bta-miR-874, one upregulated: bta-miR-33b) were identified as DE miRNAs in unrecovered FRDD cattle at W0, while bta-miR-339a was the only miRNA with a significantly downregulated expression in cattle unrecovered from FRDD at W1 (Table 3). One miRNA, bta-miR-1246, was upregulated in unrecovered TTN cattle at W1, while expressions of the other three (one upregulated: bta-miR-9-5p; two downregulated: bta-miR-296-3p and bta-miR-6523a) miRNAs were altered in TTN unrecovered cattle at W2 (Table 3). Furthermore, functional predictions of these nine DE miRNAs revealed that functions (FDR < 0.05) were enriched for four miRNAs (bta-miR-1, bta-miR-1246, bta-miR-2903, and bta-miR-33b) including transcription regulation, Zinc-finger, homeobox, and kinase functions, respectively (Table 3).

**Table 3:** DE miRNAs and enriched functions in unrecovered cattle compared to the recovered among various lameness phenotypes from W0 to W2

UNR vs. RE	Time point	miRNA	Enriched functional category	log2 FC	P value for DE miRNA
DD	W0	bta-miR-1	Transcription regulation; Transcription; Nucleus; DNA-binding	3.89	0.009
FRDD	W0	bta-miR-2903	Homeobox, conserved site; Homeodomain; Homeobox; Homeodomain-like; HOX	-2.23	0.009
		bta-miR-874	N/A	-1.05	0.009
		bta-miR-33b	Protein kinase-like domain; Protein kinase, catalytic domain; ATP binding; Serine/threonine-protein kinase, active site; Protein kinase, ATP binding site	1.58	0.038
FRDD	W1	bta-miR-339a	N/A	-6.37	0.003
TTN	W1	bta-miR-1246	Zinc-finger; Zinc; Metal-binding	7.68	< 0.001
TTN	W2	bta-miR-9-5p	N/A	1.78	< 0.001
		bta-miR-296-3p	N/A	-1.17	0.016
		bta-miR-6523a	N/A	-1.31	0.025

### Temporal changes of miRNA profiles between recovered and unrecovered cattle diagnosed with different causes of lameness

Certain cattle were responsive to corresponding treatments and recovered from lameness with a decreased gait score between W0 and W2 (defined as Recovered, RE), while several cattle did not respond to treatment and had increased gait scores and alterations in locomotion, designated as Unrecovered (UNR). Consequently, samples collected from DD, FRDD, and TTN between W0 and W2 were categorized into DDRE, DDUNR, FRDDRE, FRDDUNR, TTNRE, and TTNUNR groups based on the outcome of lameness treatment.

The changing miRNA trends between two consecutive time points (*i.e*. W0 and W1) were classified into three types: up-regulated trend (U), no significant difference trend (N), and downregulated trend (D). Hence, the overall miRNA changing patterns from W0 to W2 were defined into nine categories: D-D, D-N, D-U, N-D, N-U, N–N, U-D, U-N, and U-U. A total of eight miRNA-changing patterns were detected in the blood of RE and UNR cattle with different lameness etiologies (Table 4). The N-D and N-U patterns were detected in all groups, while no N–N changing pattern was identified in any group (Table 4). The D-D and U-D patterns were only detected for unrecovered DD cattle compared to the recovered DD (Table 4). Similarly, the U-N pattern was detected in unrecovered TTN cattle compared to the recovered TTN, and the U-D pattern was only detected in unrecovered FRDD cattle compared to the recovered FRDD (Table 4). Among each miRNA changing pattern, a range of one to eight signature miRNAs were identified (Table 4). Furthermore, signature miRNAs from five patterns were able to enrich observable functions in both RE and UNR cattle: D-D and U-D patterns in unrecovered DD cattle; the D-U pattern in recovered DD cattle; the U-N pattern in uncovered TTN cattle; the U-D pattern in unrecovered FRDD cattle (Table 5). Out of these enriched functions, the BTB/POZ-like domain was the most enriched function (enriched by the D-D and U-D patterns in unrecovered DD cattle, the D-U pattern in recovered DD cattle, and the U-N pattern in unrecovered TTN cattle) followed by Zinc-finger function (enriched by the U-D pattern in unrecovered DD cattle and U-N pattern in unrecovered TTN cattle, Table 5).

**Table 4:**
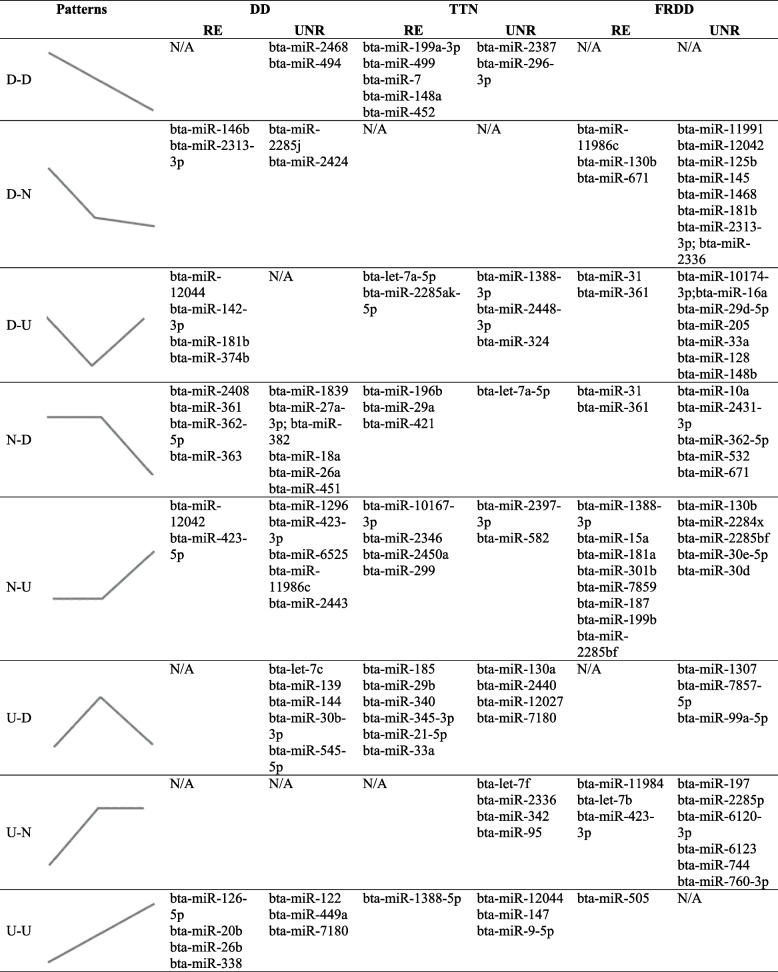
Temporal changing patterns and signature miRNAs identified from each changing pattern between recovered and unrecovered cattle with different lameness types from W0 to W2

**Table 5:** Significantly enriched functions of signature miRNAs identified from temporal changing patterns between RE and UNR cattle using DAVID

Changing patterns	Lameness type	Recovery status	Signature miRNAs	Cluster #	Predicted functions	Counts	FDR
D-D	DD	Unrecovered	bta-miR-2468 bta-miR-494	Cluster 1	Ras signaling pathway	62	2.00E-05
					Rap1 signaling pathway	58	2.00E-05
					Melanoma	25	4.70E-04
				Cluster 2	BTB/POZ fold	52	4.10E-03
					BTB/POZ-like	49	4.10E-03
					BTB	47	4.60E-02
D-U	DD	Recovered	bta-miR-12044	Cluster 1	BTB/POZ-like	48	1.10E-02
			bta-miR-142-3p		BTB/POZ fold	49	1.50E-02
			bta-miR-181b		BTB	47	5.20E-02
			bta-miR-374b				
U-D	DD	Unrecovered	bta-let-7c bta-miR-139 bta-miR-144 bta-miR-30b-3p bta-miR-545-5p	Cluster 1	BTB/POZ-like	53	8.40E-05
					BTB/POZ fold	55	1.20E-04
					BTB	53	7.40E-03
				Cluster 2	Zinc finger, C2H2	121	6.90E-05
					metal ion binding	238	3.10E-04
					Zinc finger, C2H2-like	110	6.70E-04
					Zinc finger C2H2-type/integrase DNA-binding domain	95	4.30E-03
					nucleic acid binding	105	4.40E-02
					ZnF_C2H2	110	3.20E-01
U-N	TTN	Unrecovered	bta-let-7f bta-miR-2336 bta-miR-342 bta-miR-95	Cluster 1	BTB/POZ fold	43	8.10E-04
					BTB/POZ-like	41	8.10E-04
					BTB	39	7.50E-02
				Cluster 2	Zinc finger, C2H2	87	5.30E-03
					Zinc finger, C2H2-like	82	7.10E-03
					metal ion binding	167	1.30E-02
					Zinc finger C2H2-type/integrase DNA-binding domain	71	3.20E-02
					nucleic acid binding	78	9.00E-02
					ZnF_C2H2	82	4.30E-01
U-D	FRDD	Unrecovered	bta-miR-1307 bta-miR-7857-5p bta-miR-99a-5p	Cluster 1	Homeodomain, metazoa	13	4.90E-02
					Homeodomain-like	25	4.90E-02
					Homeobox, conserved site	18	5.60E-02
					Homeobox	20	4.60E-02
					Homeodomain	20	5.60E-02
					HOX	20	3.40E-01
					sequence-specific DNA binding	24	9.00E-01
					DNA-binding	39	1.00E + 00
				Cluster 2	Wnt signaling pathway	16	6.40E-03
					Signaling pathways regulating pluripotency of stem cells	14	5.40E-02
					Hippo signaling pathway	11	8.50E-01
					Basal cell carcinoma	6	8.50E-01

### Predictiveness of selected miRNAs for lameness phenotype differentiation and lameness recovery

**Fig. 4:**
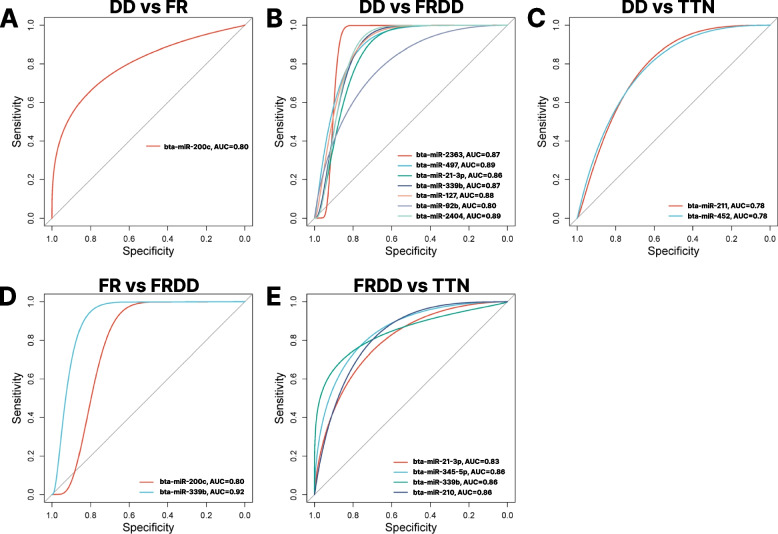
The ROC plot showing diagnostic miRNAs for DD vs FR (**A**), DD vs FRDD (**B**), DD vs TTN (**C**), FR vs FRDD (**D**), FRDD vs TTN (**E**) with good to excellent predictiveness for lameness differentiation

The DE miRNA, bta-miR-200c, showed a good power (Value = 0.8) to differentiate DD and FR (Fig. 4A, Table S2), while seven DE miRNAs (bta-miR-2363, bta-miR-497, bta-miR-21-3p, bta-miR-339b, bta-miR-127, bta-miR-92b and bta-miR-2404) were identified with good power (Value >  = 0.8) to differentiate DD and FRDD (Fig. 4B, Table S2). Furthermore, only two DE miRNAs (bta-miR-452 and bta-miR-211) showed a trend for good power to differentiate DD and TTN (Fig. 4C, Table S2). Additionally, two DE miRNAs (bta-miR-200c and bta-miR-339b) showed good to excellent power to differentiate FR and FRDD (Fig. 4D, Table S2). Another four DE miRNAs (bta-miR-21-3p, bta-miR-345-5p, bta-miR-339b, and bta-miR-210) were identified with good power to differentiate FRDD and TTN (Fig. 4E, Table S2), however, no DE miRNAs with good or excellent power was identified to differentiate FR and TTN (Table S2).

**Fig. 5:**
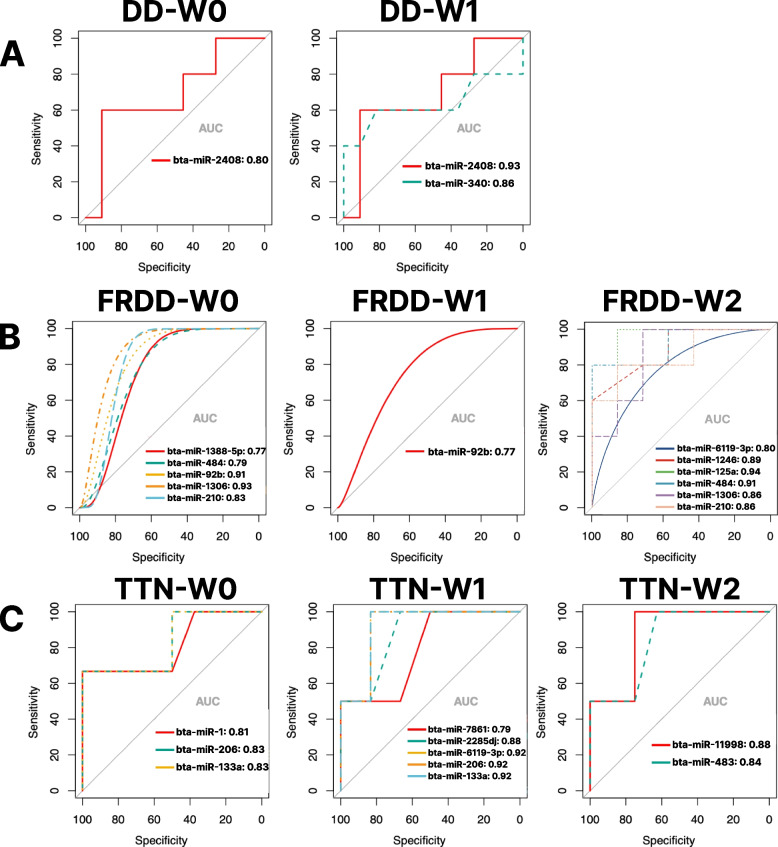
The ROC plot showing DE miRNAs with good to excellent predictiveness for lameness recovery for DD (**A**), FRDD (**B**), and TTN (**C**) from W0 to W2

In DD cattle, only one DE miRNA, bta-miR-2408, showed a good predictiveness (0.8) for DD recovery at W0 (Fig. 5A, Table S3). While at W1, the DE miRNA, bta-miR-2408, had an excellent predictiveness (0.93) for DD recovery followed by the DE miRNA, bta-miR-340 (0.86, Fig. 5A, Table S3). Additionally, for FRDD cattle at W0, the DE miRNAs, bta-miR-1306 and bta-miR-92b, exhibited excellent predictiveness (0.93, 0.91, respectively) for FRDD recovery, followed by a good and a trend of good predictiveness by bta-miR-210 (DE miRNA, 0.83), bta-miR-484 (DE miRNA, 0.79), and bta-miR-1388-5p (FRDD-specific miRNA, 0.77, Fig. 5B, Table S3), respectively. Only the DE miRNA, bta-miR-92b, had a trend of good predictiveness for FRDD recovery at W1, while eight out of 17 miRNAs (one FRDD-specific and seven DE miRNAs) showed a good to excellent predictiveness for FRDD recovery (Fig. 5B, Table S3). Moreover, three out of ten miRNAs (all of the DE miRNAs, bta-miR-1, bta-miR-206, and bta-miR-133a) presented good predictiveness (> 0.8) for TTN at W0, while nine out of ten miRNAs (three TTN-specific and six DE miRNAs) showed good to excellent predictiveness for TTN recovery at W1 (Fig. 5C, Table S3). Out of ten miRNAs at W2 in TTN cattle, only three (two TTN-specific: bta-miR-11998, bta-miR-483; one DE miRNA: bta-miR-2408) showed good to excellent predictiveness for TTN recovery (Fig. 5C, Table S3).

### Validation of miRNA expressions using reverse transcription-quantitative real-time PCR

**Fig. 6:**
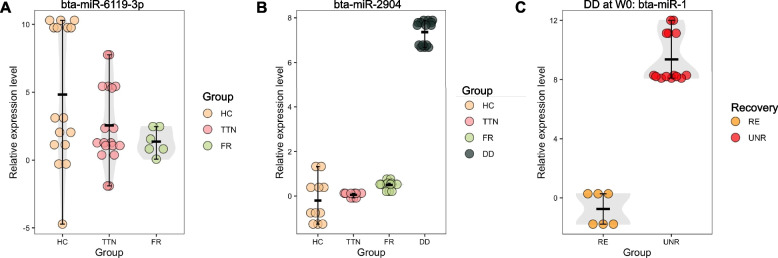
Validations of expression levels of selected miRNAs using RT-qPCR. One DE miRNA bta-miR-6119-3p (**A**), one phenotype-specific miRNA (**B**), and one DE miRNA for recovered and recovered cattle (**C**) were subjected to RT-qPCR, respectively. The healthy cattle without lameness (HC or Recovered) were considered as the reference group for analysis. The value of the y-axis represents the relative expression level computed by the ΔΔCt method

One DE miRNA bta-miR-6119-3p (FR, TTN vs HC), one phenotype-specific miRNA (bta-miR-2904 as DD-specific), and one miRNA bta-miR-6119-3p identified for unrecovered and recovered DD cattle were subjected to RT-qPCR. The RT-qPCR results were consistent with the RNA-seq-based identifications (Fig. 6). By using RT-qPCR, we verified that downregulated DE miRNA bta-miR-6119-3p has significantly lower expression levels in TTN and FR cattle compared to healthy cattle (P < 0.05, Fig. 6A). The phenotype-specific bta-miR-2904 had a significantly higher expression level in DD, however, such expression tended to 0 among other phenotypes (Fig. 6B). Besides, the expression of bta-miR-1 was higher in unrecovered DD cattle compared to recovered, which was consistent with miRNAome results (Fig. 6C).

## Discussion

Our study comprehensively assessed miRNA profiles and the changing patterns in response to various lameness phenotypes and lameness recovery with the aim to determine if miRNAs could serve as potential diagnostic markers for different lameness phenotypes and recovery in feedlot cattle. The number of expressed miRNAs in the whole blood of feedlot steers (321 miRNAs) was similar to previous bovine blood miRNA profiling (320 miRNAs) in the whole blood of beef cattle [28]. Among these identified miRNAs, the top ten most expressed miRNAs were similar to those reported in cattle sera and exosome, with bta-miR-486 also being the most abundant [27, 29], suggesting that most expressed miRNAs in bovine blood are highly consistent.

Our current study revealed that changes in miRNA expression were related to physiological changes in animals, suggesting that the blood miRNA expressions may be associated with mechanisms of pathogenesis of different lameness phenotypes. The bta-miR-6119-3p was the only downregulated miRNA in all lameness phenotypes pre-treatment with its predicted functions of the Zinc-finger domain. The Zinc-finger domain is one of the most frequently utilized DNA-binding motifs and belongs to transcriptional factors that regulate various cellular processes for the maintenance of host homeostasis [30, 31]. It has been reported that Zinc-finger protein family members, such as *ZNF750*, were related to skin diseases and the pathogenesis of seborrhea-like dermatitis and psoriasis in humans [32] and the promotion of *ZNF750* is potentially related to skin diseases. Since miRNAs are highly conserved between different species and the predicted function of bta-miR-6119-3p is related to the zinc-finger domain, it is speculated that this miRNA may have similar regulatory roles in skin diseases, including dermatitis on the digital cushion of cattle hoofs. As one of the most abundant transcription factors, zinc-finger proteins also play essential roles in muscle differentiation [31]. The decreased *SMYD1*, one of the Zinc-finger proteins, has been reported to reduce the muscle cell myofiber formation and depress the muscle’s special gene expression [33]. In lame cattle, the muscle development function was affected due to restricted movement and reduced feed intake. Thus, the differential expression of bta-miR-6119-3p may be related to the muscle degeneration caused by different phenotypes of lameness. Furthermore, functional analysis revealed that the expression of this miRNA was decreased with enhanced functions including the formation of dermatitis and epidermis in lame cattle. It has been reported that bta-miR-6119-3p is capable of triggering host interferons in response to viral infections in cattle diagnosed with foot mouth disease and bovine respiratory disease [34, 35], suggesting the potential role of this miRNA in host immune response regulation. Consequently, the lower expression level of bta-miR-6119-3p in all lameness phenotypes may suggest an active miRNA-mediated inflammatory response during tissue damage. Many of the other downregulated DE miRNAs in lame cattle were predicted to play a role in the inhibition of cell growth. For example, miR-340 (downregulated in DD cattle), known for its involvement in restraining the growth of colorectal cancer cells, showed significantly reduced expression in colon tissue cells from individuals who survived more than 5 years after a colon cancer diagnosis [36]. Likewise, decreased expression levels of these DE miRNAs in lameness cattle could suggest he reduced inhibited functions of cell growth, potentially facilitating cell regeneration and tissue recovery in the foot and/or toe after lameness. Further studies are needed to verify the regulatory mechanisms of downregulated DE miRNAs in lameness.

Notably, several upregulated DE miRNAs (bta-miR-1, -206, and -133a) among all lameness phenotypes were identified as muscle-specific in previous animal studies [37–40]. The functions of these muscle-specific miRNAs relate to skeletal muscle development and progression [37–40]. In our study, muscle-specific miRNAs were found in the blood, likely because the expression levels of these miRNAs may have been elevated in response to lameness and were released from muscles into the blood through exosomes [41]. This release could ultimately contribute to muscle atrophy due to reduced physical activity. Moreover, the predicted functions of the upregulated DE miRNAs in the blood of lame cattle were involved in traumatic muscle stress [42] and intramuscular adipogenesis [43], further suggesting the involvement of miRNAs in altered body weight or muscle atrophy after lameness. Further investigations are needed to verify our speculations by examining the expressions of these miRNAs in samples from the wounds and swabs of lame cattle.

In addition to the identification of DE miRNAs between lame and healthy cattle, we observed that the expression of some miRNAs was only detected in the blood of specific lameness phenotypes. It is notable that many of the lameness phenotype-specific miRNAs had low expression and had no predicted functions. However, it was reported that some lameness phenotype-specific miRNAs were differentially expressed in the cattle blood under different physiological conditions, which provides evidence of their potential regulatory role in the corresponding conditions. For example, the DD phenotype-specific miRNA, bta-miR-2904, has been identified to be differentially expressed in bovine milk under inflammation stress and in the bovine granulosa cells of *Staphylococcus aureus* challenged cattle [44]. For FR-specific miRNAs, bta-miR-200a has been found to be upregulated in mastitis and in systemic lupus erythematosus models [45]. Wang et al. reported that the expression of bta-miR-2887 was downregulated in the intestinal tissue of cattle with *Escherichia coli* O157:H7 fecal shedding and was involved in hematological system development [46]. Although our study did not identify enriched functions of phenotype-specific miRNAs, previous literature suggests that phenotype-specific miRNAs may have target genes or related functions involved in immune and infection responses. Although the expressions of phenotype-specific miRNAs may be due to their response to different lameness phenotypes, further research is needed to identify target genes for the selected DE and phenotype-specific miRNAs to validate their roles in lameness pathogenesis.

Previous miRNAome studies focused more on differentially expressed miRNAs, *i.e.* the identification of a total of 14 differentially expressed miRNAs such as bta-let-7f, bta-let-7c, bta-miR-30c, bta-miR-101, bta-miR-26a, bta-miR-205 and bta-miR-143 on Day 60 relative to Day 0 of pregnancy in dairy cattle [47]. However, there is a lack of longitudinal observations of miRNAomes under specific phenotypes for identification of time/age specific miRNAs. Our study is the first to report the temporal miRNA expression in the same cattle in relation to their diagnosis and recovery of lameness. The expression of time-point specific miRNAs is important since they may represent host physiological responses to lameness at different stages of recovery, and the functions of certain time point-specific miRNAs may relate to lameness pathogenesis. For instance, bta-miR-149-5p was found at W1 in DD and at W2 in FRDD cattle with predicted functions as a negative regulator of adipocyte differentiation in bovine primary preadipocytes [48, 49]. Meanwhile, bta-miR-30f was considered a W2-specific miRNA in FRDD, acting as a lipid deposit regulator in cattle, which negatively affects the deposition of fat by inhibiting the PPAR signaling pathway [50]. Previous studies identified these miRNAs (bta-miR-149-5p and bta-miR-30f) with predicted functions associated with adipogenesis in beef cattle [49, 50]. Few studies focus on how adipogenesis progression is related to lameness and the role of time-specific miRNAs during such interactions. Previous studies identified that adipose tissue-derived mesenchymal stem cells could exert therapeutic effects on canine lameness [51, 52], suggesting the importance of adipogenesis and fat deposit during lameness. Our study revealed that the potential role of time-specific miRNAs was involved in adipogenesis during lameness. A possible explanation of such altered adipogenesis function may relate to the reduced feed intake of lame cattle which could lead to a decrease in the body fat deposit. Therefore, these identified time point-specific miRNAs suggest the potential regulatory role for lameness-related adipogenesis; however, further studies are needed to verify the functions of identified time-point specific miRNAs in regulating lameness through adipogenesis.

On-farm lameness is often diagnosed by veterinarians, farmers, and hoof trimmers via visual assessments. However, cattle could manifest comparable clinical signs of different lameness phenotypes, which delayed the efficient lameness phenotyping and the subsequent treatments [12]. Therefore, exploring the potential molecular mechanisms of miRNAs affecting lameness in lame cattle and recovered cattle could further our understanding of the involvement of miRNAs in lameness. Our study identified a higher expression of bta-miR-1246 in unrecovered TTN cattle at W1 post-treatment. The increased expression of this miRNA was reported in *S. aureus* infected lame chickens [53–55] and was considered a regulator for host inflammation and immune responses [56, 57]. Therefore, the identified bta-miR-1246 in TTN unrecovered cattle suggests that the occurrence of TTN could be due to bacterial infection [58], leading to inhibited host immune responses in unrecovered TTN cattle. Additionally, our study identified that miRNAs, such as bta-miR-2468 and bta-miR-494, exhibited downregulated expression patterns over the two-week treatment in unrecovered DD cattle. The bta-miR-494 plays an essential role in the response to gram-negative bacterial infections [59], as shown in the significantly reduced expressions in mouse lungs after the lipopolysaccharide (LPS) challenge [60]. It is known that primary pathogens of lameness phenotypes are gram-negative bacteria (e.g. *Spirochete* and *Fusobacterium*) with the ability to produce LPS and cause host inflammation [61, 62]. Therefore, the identified bta-miR-494 could play an essential role in regulating DD occurrence by interacting with gram-negative bacteria and could be involved in DD-related inflammation in unrecovered cattle. Interestingly, the bta-let-7 family was identified in both unrecovered TTN and DD cattle. This family was associated with host immune response suppression and is negatively regulated by the level of anti-inflammatory cytokines in cattle blood [60] and is involved in the regulation of skeletal muscle development [63]. In a human study, the upregulation of the bta-let-7 family was reported as a marker of cell cycle function damage, which may reduce the renewal and regeneration of muscle cells in older individuals [64]. Thus, the up-regulation of the bta-let-7 family in the first week after treatment in unrecovered TTN and DD cattle may contribute to suppressed immune functions and reduced muscle cell repair. Taken together, these findings suggest that unrecovered cattle that were not responsive to treatment may suffer from intense inflammation due to inhibited host immune responses and a decreased ability for tissue repairment. However, the sample size used for the diagnosis of lameness recovery is relatively small for certain lameness phenotype, a larger cohort together with in vivo and in vitro studies are needed to verify miRNAome-regulated mechanisms.

Our study further assessed the abilities of phenotype DE miRNAs serving as potential markers to differentiate lameness phenotypes with the aim of providing directions for efficient on-farm lameness detection. Particularly, one DE miRNA bta-miR-339b showed a good to excellent power to differentiate DD vs FRDD, FR vs FRDD, as well as FRDD vs TTN, and several miRNAs with good power to differentiate two different lameness phenotypes. One study characterized the critical role of bta-miR-339b, which exhibited notable negative correlations with enzootic bovine leukosis proviral load in cattle, suggesting this miRNA is a potential regulator for viral infections [65]. Although there are limited studies on the mechanisms of bta-miR-339b during lameness pathogenesis and in other diseases, we speculate that bta-miR-339b, together with other potential miRNA markers, could correspond to different lameness phenotypes. Further studies are needed to verify if and how combinations of miRNA markers could be effective to differentiate various lameness phenotypes. These results suggest that DE miRNAs were not only involved in mechanisms of lameness but also had the potential as diagnostic markers for lameness phenotyping before treatment. Moreover, both phenotype specific and DE miRNAs identified before treatment in lame cattle compared to healthy controls showed good to excellent predictiveness for lameness recovery at W0 to W2. However, the predictiveness of certain DE miRNAs (*i.e.* bta-miR-11998) was 1 (that means this miRNA can always make a correct prediction), which may be due to the limited samples used for each time point. The function of bta-miR-11998 has not been studied, if and how this miRNA plays the regulatory role in lameness phenotypes is also unclear. Further explorations are needed to verify the role of this miRNA in lameness progression. Regardless, our study demonstrates the potential of blood miRNAs serving as markers for phenotyping and lameness recovery prediction, facilitating timely treatment. However, future studies using a large cohort of feedlot cattle for verification and the combination of various miRNAs for lameness predictions are needed.

## Conclusions

In conclusion, our study investigated the potential regulatory roles of miRNAomes in the pathogenesis and recovery of lame cattle. Differentially expressed (DE) miRNAs in lame cattle harbor distinct functions including Zinc-finger and muscle cell regulations that are primarily associated with lameness pathogenesis. Phenotype-specific miRNAs were identified to be associated with specific mechanisms of each lameness. Additionally, blood miRNA expression patterns contribute to the recovery of different lameness phenotypes with functions of these miRNAs relating to inhibited host immune responses and decreased muscular functions. Furthermore, our study highlights that phenotype-specific and DE miRNAs can serve as powerful diagnostic markers to differentiate various lameness phenotypes as well as the prediction of lameness recovery, shedding light on accurate on-farm lameness detection and phenotyping methods. However, future studies using a large cohort of feedlot cattle are needed to verify the regulatory role of miRNAs in lameness and the prediction accuracy of identified miRNA markers.

## Supplementary Information

Supplementary Material 1.

Supplementary Material 2.

## Data Availability

All the sequencing data used in the current study has been submitted to NCBI Sequence Read Archive (SRA) under the accession numbers PRJNA1044093.

## Abbreviations

ALDS: Automated lameness detection systems
AUC: Area under the ROC curve
circ-miRNAs: Circular miRNAs
D: The downregulated trend
DAVID: Database for Annotation, Visualization and Integrated Discovery
DD: Digital dermatitis
DE: Differentially expressed
FCM: Fuzz C-Means clustering
FDR: False discovery rate
FR: Foot rot
FRDD: FR combined DD
LPS: Lipopolysaccharide
Sec: Seconds
Min: Minutes
mRNA: Messenger RNA
miRNAs: MicroRNAs
N: No significantly different trend
RE: Recovered
RIN: RNA Integrity Number
ROC: Receiver Operating Characteristics
RPM: Reads per million mapped reads
TTN: Toe tip necrosis
U: The up-regulated trend
UNR: Unrecovered
W0: Week 0
W1: Week 1
W2: Week 2
