# Supplementary figures and images for: Profiling of blood miRNAomes revealed the potential regulatory role of miRNAs in various lameness phenotypes in feedlot cattle
Source: BMC Genomics. 2024 Dec 18;25:1190. doi: 10.1186/s12864-024-10807-z (PMC11653651; doi:10.1186/s12864-024-10807-z)

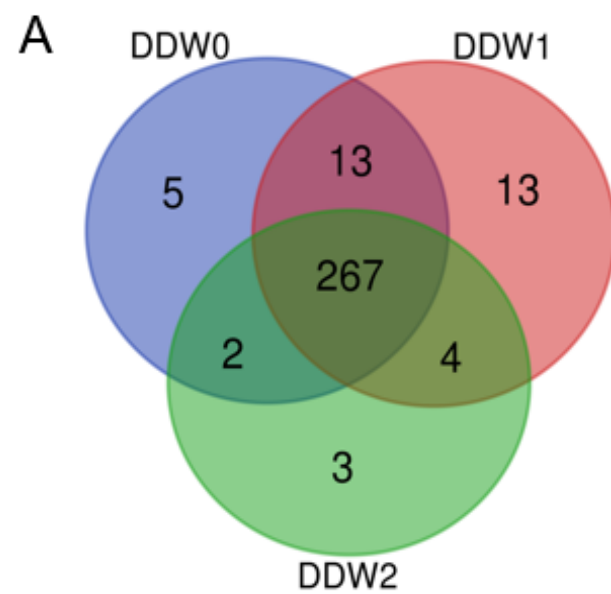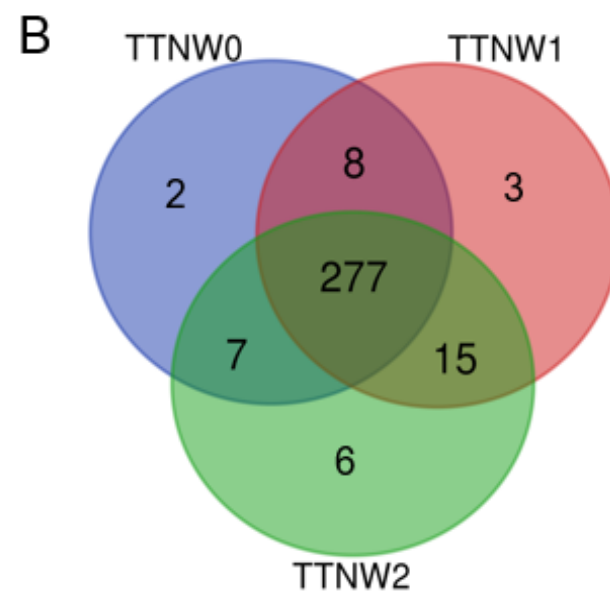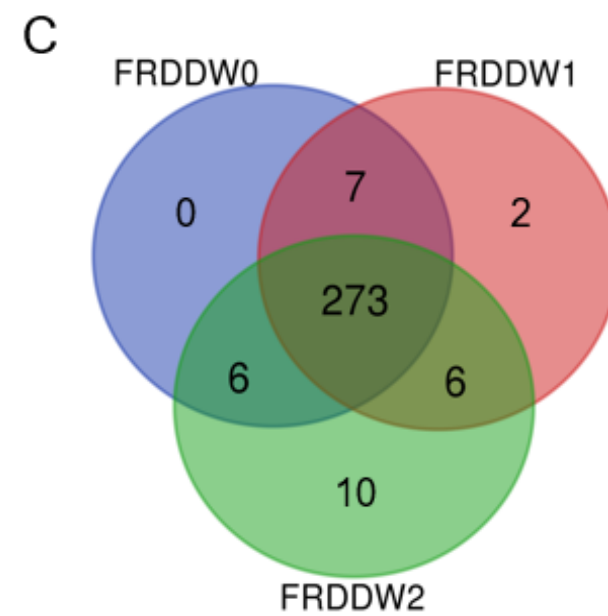

Supplement: Supplementary file 1 — Supplementary Material 1. [file 12864_2024_10807_MOESM1_ESM.pdf]
