# Supplementary material for: Profiling of blood miRNAomes revealed the potential regulatory role of miRNAs in various lameness phenotypes in feedlot cattle
Source: BMC Genomics. 2024 Dec 18;25:1190. doi: 10.1186/s12864-024-10807-z (PMC11653651; doi:10.1186/s12864-024-10807-z)
Supplement: Supplementary file 2 — Supplementary Material 2. [file 12864_2024_10807_MOESM2_ESM.docx]

**Table S1. Identified differential expressed miRNAs among different lameness phenotypes**

| **Group** | **DE miRNA** | **log2FoldChange** | ***P*adj** |
| --- | --- | --- | --- |
| DD vs FR | bta-miR-133a | -1.44 | 1.41E-02 |
|  | bta-miR-141 | -1.31 | 7.48E-05 |
|  | bta-miR-200c | -1.12 | 7.39E-09 |
|  | bta-miR-206 | -1.06 | 3.43E-02 |
|  | bta-miR-122 | 1.6 | 5.27E-04 |
| DD vs FRDD | bta-miR-2363 | -1.76 | 7.97E-03 |
|  | bta-miR-497 | -1.23 | 1.01E-05 |
|  | bta-miR-21-3p | -1.17 | 7.80E-05 |
|  | bta-miR-339b | -1.11 | 9.10E-07 |
|  | bta-miR-339a | -1.07 | 1.10E-06 |
|  | bta-miR-10225a | -1.07 | 9.02E-03 |
|  | bta-miR-127 | -1.01 | 5.93E-04 |
|  | bta-miR-877 | 1 | 1.84E-02 |
|  | bta-miR-2284z | 1.01 | 2.47E-07 |
|  | bta-miR-374b | 1.01 | 8.88E-08 |
|  | bta-miR-92a | 1.04 | 1.04E-05 |
|  | bta-miR-2284aa | 1.06 | 1.13E-07 |
|  | bta-miR-320a | 1.2 | 1.17E-04 |
|  | bta-miR-451 | 1.27 | 8.88E-08 |
|  | bta-miR-2332 | 1.28 | 9.02E-03 |
|  | bta-miR-1 | 1.36 | 3.26E-02 |
|  | bta-miR-1249 | 1.45 | 2.06E-03 |
|  | bta-miR-92b | 1.49 | 1.04E-07 |
|  | bta-miR-2404 | 1.83 | 2.59E-04 |
|  | bta-miR-1434-3p | 1.93 | 1.90E-03 |
| DD vs TTN | bta-miR-133a | -3.34 | 8.05E-07 |
|  | bta-miR-206 | -2.19 | 9.61E-04 |
|  | bta-miR-211 | -1.32 | 2.36E-02 |
|  | bta-miR-452 | -1.29 | 2.30E-02 |
|  | bta-miR-2285bf | 1.03 | 1.39E-02 |
|  | bta-miR-129-5p | 1.17 | 3.43E-02 |
| FR vs FRDD | bta-miR-2363 | -1.7 | 4.08E-03 |
|  | bta-miR-199a-5p | -1.2 | 6.72E-03 |
|  | bta-miR-127 | -1.18 | 6.69E-06 |
|  | bta-miR-339b | -1.18 | 1.18E-08 |
|  | bta-miR-339a | -1.14 | 1.18E-08 |
|  | bta-miR-21-5p | 1.02 | 8.32E-12 |
|  | bta-miR-877 | 1.1 | 4.95E-03 |
|  | bta-miR-92b | 1.18 | 6.67E-06 |
|  | bta-miR-1434-3p | 1.5 | 1.19E-02 |
|  | bta-miR-200c | 1.52 | 5.49E-13 |
|  | bta-miR-206 | 1.75 | 3.40E-04 |
|  | bta-miR-133a | 1.83 | 1.66E-03 |
| FR vs TTN | bta-miR-133a | -1.9 | 1.77E-02 |
|  | bta-miR-1 | -1.8 | 1.98E-02 |
|  | bta-miR-122 | -1.39 | 3.95E-02 |
|  | bta-miR-11986c | 1.02 | 3.78E-02 |
|  | bta-miR-141 | 1.19 | 1.77E-02 |
|  | bta-miR-129 | 1.42 | 1.99E-02 |
| FRDD vs TTN | bta-miR-129 | -1.51 | 5.39E-03 |
|  | bta-miR-21-3p | -1.15 | 2.15E-03 |
|  | bta-miR-11986c | -1.13 | 1.60E-02 |
|  | bta-miR-345-5p | -1.09 | 1.15E-03 |
|  | bta-miR-339b | -1.08 | 1.54E-04 |
|  | bta-miR-210 | -1.08 | 5.59E-06 |
|  | bta-miR-339a | -1.01 | 2.56E-04 |
|  | bta-miR-92b | 1.05 | 3.62E-03 |
|  | bta-miR-2404 | 1.62 | 1.10E-02 |
|  | bta-miR-211 | 1.8 | 5.18E-04 |
|  | bta-miR-1434-3p | 2.18 | 4.15E-03 |
|  | bta-miR-1 | 2.64 | 2.80E-04 |
|  | bta-miR-206 | 2.88 | 5.59E-06 |
|  | bta-miR-133a | 3.73 | 2.71E-07 |

**Table S2. The predictiveness of between-group DE miRNAs for lameness phenotype differentiation**

| **Group** | **DE miRNA** | **The value of Area under AOC curve** |
| --- | --- | --- |
| DD vs FR | bta-miR-133a | 0.74 |
|  | bta-miR-141 | 0.78 |
|  | bta-miR-200c | 0.80 |
|  | bta-miR-206 | 0.72 |
|  | bta-miR-122 | 0.66 |
| DD vs FRDD | bta-miR-2363 | 0.87 |
|  | bta-miR-497 | 0.89 |
|  | bta-miR-21-3p | 0.86 |
|  | bta-miR-339b | 0.87 |
|  | bta-miR-339a | 0.62 |
|  | bta-miR-10225a | 0.77 |
|  | bta-miR-127 | 0.88 |
|  | bta-miR-877 | 0.58 |
|  | bta-miR-2284z | 0.63 |
|  | bta-miR-374b | 0.64 |
|  | bta-miR-92a | 0.67 |
|  | bta-miR-2284aa | 0.45 |
|  | bta-miR-320a | 0.66 |
|  | bta-miR-451 | 0.75 |
|  | bta-miR-2332 | 0.65 |
|  | bta-miR-1 | 0.67 |
|  | bta-miR-1249 | 0.68 |
|  | bta-miR-92b | 0.80 |
|  | bta-miR-2404 | 0.89 |
|  | bta-miR-1434-3p | 0.72 |
| DD vs TTN | bta-miR-133a | 0.71 |
|  | bta-miR-206 | 0.65 |
|  | bta-miR-211 | 0.78 |
|  | bta-miR-452 | 0.78 |
|  | bta-miR-2285bf | 0.68 |
|  | bta-miR-129-5p | 0.62 |
| FR vs FRDD | bta-miR-2363 | 0.78 |
|  | bta-miR-199a-5p | 0.79 |
|  | bta-miR-127 | 0.53 |
|  | bta-miR-339b | 0.92 |
|  | bta-miR-339a | 0.63 |
|  | bta-miR-21-5p | 0.71 |
|  | bta-miR-877 | 0.66 |
|  | bta-miR-92b | 0.68 |
|  | bta-miR-1434-3p | 0.59 |
|  | bta-miR-200c | 0.80 |
|  | bta-miR-206 | 0.78 |
|  | bta-miR-133a | 0.72 |
| FR vs TTN | bta-miR-133a | 0.52 |
|  | bta-miR-1 | 0.53 |
|  | bta-miR-122 | 0.64 |
|  | bta-miR-11986c | 0.67 |
|  | bta-miR-141 | 0.68 |
|  | bta-miR-129 | 0.74 |
| FRDD vs TTN | bta-miR-129 | 0.79 |
|  | bta-miR-21-3p | 0.83 |
|  | bta-miR-11986c | 0.76 |
|  | bta-miR-345-5p | 0.86 |
|  | bta-miR-339b | 0.86 |
|  | bta-miR-210 | 0.86 |
|  | bta-miR-339a | 0.42 |
|  | bta-miR-92b | 0.70 |
|  | bta-miR-2404 | 0.78 |
|  | bta-miR-211 | 0.78 |
|  | bta-miR-1434-3p | 0.77 |
|  | bta-miR-1 | 0.55 |
|  | bta-miR-206 | 0.73 |
|  | bta-miR-133a | 0.65 |

**Table S3. Predictiveness of pre-treatment phenotype-specific and DE miRNAs for lameness recovery**

| miRNAs | Role of miRNAs | W0 | W1 | W2 | Lameness type |
| --- | --- | --- | --- | --- | --- |
| bta-miR-2887 | DD-specific | 0.76 | 0.64 | 0.67 | DD |
| bta-miR-2320-3p | DD-specific | 0.55 | 0.69 | 0.61 | DD |
| bta-miR-2904 | DD-specific | 0.70 | 0.53 | 0.59 | DD |
| bta-miR-6119-3p | DE miRNA | 0.50 | 0.58 | 0.55 | DD |
| bta-miR-2408 | DE miRNA | 0.80 | 0.93 | 0.70 | DD |
| bta-miR-340 | DE miRNA | 0.58 | 0.86 | 0.66 | DD |
| bta-miR-1388-5p | FRDD-specific | 0.77 | 0.54 | 1.00 | FRDD |
| bta-miR-494 | FRDD-specific | 0.57 | 0.47 | 0.47 | FRDD |
| bta-miR-2483-5p | FRDD-specific | 0.49 | N/A | N/A | FRDD |
| bta-miR-6119-3p | DE miRNA | 0.57 | N/A | 0.80 | FRDD |
| bta-miR-1434-3p | DE miRNA | 0.48 | 0.57 | 0.67 | FRDD |
| bta-miR-211 | DE miRNA | 0.61 | 0.49 | 0.69 | FRDD |
| bta-miR-1246 | DE miRNA | 0.45 | N/A | 0.89 | FRDD |
| bta-miR-125a | DE miRNA | 0.62 | 0.64 | 0.94 | FRDD |
| bta-miR-484 | DE miRNA | 0.79 | N/A | 0.91 | FRDD |
| bta-miR-92b | DE miRNA | 0.91 | 0.77 | 1.00 | FRDD |
| bta-miR-1306 | DE miRNA | 0.93 | N/A | 0.86 | FRDD |
| bta-miR-2378 | DE miRNA | N/A | N/A | N/A | FRDD |
| bta-miR-338 | DE miRNA | 0.70 | 0.66 | 0.54 | FRDD |
| bta-miR-497 | DE miRNA | 0.63 | N/A | 0.57 | FRDD |
| bta-miR-210 | DE miRNA | 0.83 | 0.57 | 0.86 | FRDD |
| bta-miR-345-5p | DE miRNA | 0.63 | 0.46 | 0.63 | FRDD |
| bta-miR-21-3p | DE miRNA | 0.52 | N/A | 0.51 | FRDD |
| bta-miR-7861 | TTN-specific | 0.67 | 0.79 | 0.69 | TTN |
| bta-miR-11998 | TTN-specific | 0.42 | 1.00 | 0.88 | TTN |
| bta-miR-483 | TTN-specific | 0.52 | 0.50 | 0.84 | TTN |
| bta-miR-2285dj | TTN-specific | 0.63 | 0.88 | 0.47 | TTN |
| bta-miR-6119-3p | DE miRNA | 0.67 | 0.92 | 0.56 | TTN |
| bta-miR-2408 | DE miRNA | 0.65 | 1.00 | 1.00 | TTN |
| bta-miR-340 | DE miRNA | 0.63 | 1.00 | 0.69 | TTN |
| bta-miR-1 | DE miRNA | 0.81 | 1.00 | 0.63 | TTN |
| bta-miR-206 | DE miRNA | 0.83 | 0.92 | 0.56 | TTN |
| bta-miR-133a | DE miRNA | 0.83 | 0.92 | 0.50 | TTN |
